# Columnaris-causing bacteria *Flavobacterium covae* isolated from diseased fish in Australia

**DOI:** 10.1128/mra.00129-25

**Published:** 2025-04-04

**Authors:** Francisca Samsing, Roisin Sullivan, Vincenzo A. Costa, Benjamin R. LaFrentz

**Affiliations:** 1 Sydney School of Veterinary Science, The University of Sydney, Camden, New South Wales, Australia; 2 United States Department of Agriculture, Agricultural Research Service, Aquatic Animal Health Research Unit, Auburn, Alabama, USA; University of Southern California, Los Angeles, California, USA

**Keywords:** aquaculture, aquatic animal health, fish pathogens, nanopore

## Abstract

*Flavobacterium covae* is one of four *Flavobacterium* spp. causing columnaris disease in teleost fish. Here, we present the draft genome of a new isolate, FC5-24/06, which is the first reported from Australia.

## ANNOUNCEMENT

Columnaris disease is a prevalent freshwater fish disease worldwide (1–4). Historically, *Flavobacterium columnare* was the sole recognized cause (5, 6), but recent studies have reclassified it into four distinct species called columnaris-causing bacteria (CCB): *F. columnare*, *F. covae*, *F. davisii*, and *F. oreochromis* (7).

In 2024, a barramundi (*Lates calcarifer*) producer in Camden, New South Wales, Australia, reported a disease outbreak in fingerlings within a recirculating aquaculture system (RAS). Affected fish exhibited clinical signs consistent with columnaris disease, including “saddle-back” lesions, scale loss, fin rot, and increased mortality (5). Diagnostic samples from head to kidney of moribund fingerling (80 mm total length) were plated onto selective modified Shieh agar (8) and incubated at 26°C. Yellow, rhizoid, and adherent colonies, typical of CCB, were passaged three times before DNA extraction. A single colony, designated isolate FC5-24/06, was extracted using the Quick-DNA Miniprep Plus Kit (Zymo Research). Genomic DNA was quality-checked via NanoDrop 1000 and quantified with a Qubit 1.0 fluorometer. All animal procedures were approved by The University of Sydney AEC (Project 2021/1867) as part of clinical cases for teaching.

Oxford Nanopore Technology (ONT) sequencing was performed using a Minion Mk1C with the Rapid Barcoding 24 Kit V14 (SQK-RB114.24) on a R10.4.1 flow cell (FLO-MIN114). No size selection was performed prior to library preparation and sequencing. POD5 files were basecalled in MinKNOW v.24.02.8 using Dorado (dna_r10.4.1_e8.2_400bps_5khz_sup). A total of 430,379 reads were generated (mean PHRED score: Q16; median read length: 1,862 bp). Reads were filtered using chopper v.0.2.0 to retain high-quality reads (≥Q18, ≥1,000 bp), yielding 100,933 reads (mean quality score: 20.9; median read length: 2,849 bp) (9). *De novo* assembly was conducted using Flye v.2.9.3 (flye --nano-hq --meta) (10). The draft genome of FC5-24/06 consisted of four contigs, totaling 3.29 Mb (mean coverage: 74.3×, G + C%: 30.80%). The two largest contigs were 1.67 and 1.60 Mb, with two smaller contigs (8.2 and 7.3 Kb). Taxonomic classification was performed using Kraken 2 (11, 12). Default parameters were used for all software unless otherwise specified.

Comparing genomics using average nucleotide identities (ANI) with CCB reference genomes (7, 13) using fastani v1.32 (14) confirmed FC5-24/06 as *F. covae*. ANI values showed 98.9% similarity with *F. covae* strain AL-02–36^T^ (GenBank: CP067379), with lower similarity to *F. columnare* (91.3%, GenBank: PCMX01000000), *F. davisii* (85.4%, GenBank: CP067378), and *F. oreochromis* (83.4%, GenBank: CP067377). This confirms FC5-24/06 as the first *F. covae* isolate reported in Australia.

**Fig 1:**
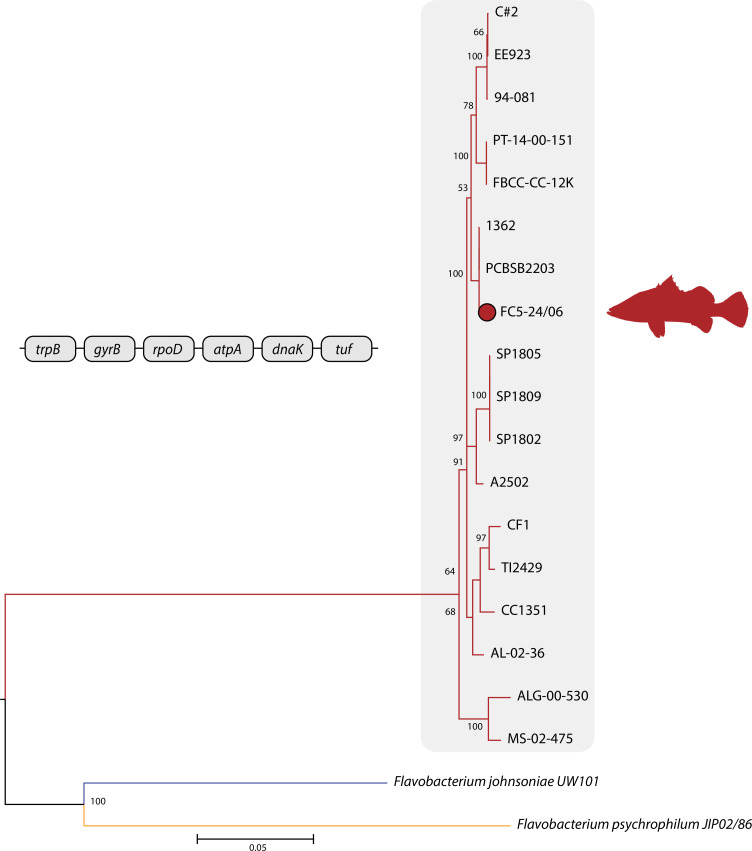
Phylogenetic tree of *Flavobacterium sp*. based on six concatenated housekeeping genes constructed using maximum likelihood method (GTR + G + I model) and rooted with *F. johnsoniae* and *F. psychrophilum*. Bootstrap values (1,000 replicates) are shown. Shaded taxa represent *F. covae*, with newly identified isolate highlighted. Scale bar represents nucleotide substitutions per site.

Genome annotation was performed using RAST v. 2.0, which identified 2,971 coding sequences (CDSs) and 31 tRNAs (15). In addition, the NCBI Prokaryotic Genome Annotation Pipeline (PGAP) automatically annotated the genome upon submission, identifying 2,787 CDSs, 78 tRNAs, and 94 RNA genes (16). Differences in gene predictions reflect variations in annotation pipelines. Multilocus sequence analysis (MLSA) of six housekeeping genes (*trpB, gyrB, rpoD*, *atpA*, *dnaK,* and *tuf*) was conducted using MEGA11 (17), including FC5-24/06, type strain AL-02–36^T^, and reference *F. covae* isolates (18, 19). Phylogenetic analysis using maximum likelihood method (GTR + G + I model) revealed FC5-24/06 clustering with two *F. covae* isolates from Thailand, one associated with diseased barramundi (PCBSB2203), but distinct from other Thai isolates (SP1802, SP1805, and SP1809) (Fig. 1).

This report documents the first *F. covae* isolate from Australia following the reclassification of *F. columnare*. Expanding genomic surveillance of CCB in different aquaculture sectors is crucial for targeted disease management and mitigating antimicrobial resistance (20).

## Data Availability

This Whole Genome Shotgun project has been deposited at GenBank under the accession JBIWYB000000000. The version described in this paper is version JBIWYB010000000. *Flavobacterium covae* FC5-24/06 is under the BioSample accession number SAMN44567531 and Bioproject number PRJNA1181634.

## References

[1] Arias CR, Welker TL, Shoemaker CA, Abernathy JW, Klesius PH. 2004. Genetic fingerprinting of Flavobacterium columnare isolates from cultured fish. J Appl Microbiol 97:421–428. doi:10.1111/j.1365-2672.2004.02314.x 15239710

[2] LaFrentz BR, Khoo LH, Lawrence ML, Petrie-Hanson L, Hanson LA, Baumgartner WA, Hemstreet WG, Kelly AM, García JC, Shelley JP, Johnston AE, Bruce TJ, Griffin MJ. 2024. Flavobacterium covae is the predominant species of columnaris-causing bacteria impacting the Channel Catfish industry in the southeastern United States. J Aquat Anim Health 36:3–15. doi:10.1002/aah.10207 37859458

[3] Tien NT, Dung TT, Tuan NA, Crumlish M. 2012. First identification of Flavobacterium columnare infection in farmed freshwater striped catfish Pangasianodon hypophthalmus. Dis Aquat Org 100:83–88. doi:10.3354/dao02478 22885517

[4] Wonmongkol P, Sukhavachana S, Ampolsak K, Srisapoome P, Suwanasopee T, Poompuang S. 2018. Genetic parameters for resistance against Flavobacterium columnare in Nile tilapia Oreochromis niloticus (Linnaeus, 1758). J Fish Dis 41:321–328. doi:10.1111/jfd.12728 29064113

[5] Declercq AM, Haesebrouck F, Van den Broeck W, Bossier P, Decostere A. 2013. Columnaris disease in fish: a review with emphasis on bacterium-host interactions. Vet Res 44:27. doi:10.1186/1297-9716-44-27 23617544 PMC3648355

[6] Wahli T, Madsen L. 2018. Flavobacteria, a never ending threat for fish: a review. Curr Clin Micro Rpt 5:26–37. doi:10.1007/s40588-018-0086-x

[7] LaFrentz BR, Králová S, Burbick CR, Alexander TL, Phillips CW, Griffin MJ, Waldbieser GC, García JC, de Alexandre Sebastião F, Soto E, Loch TP, Liles MR, Snekvik KR. 2022. The fish pathogen Flavobacterium columnare represents four distinct species: Flavobacterium columnare, Flavobacterium covae sp. nov., Flavobacterium davisii sp. nov. and Flavobacterium oreochromis sp. nov., and emended description of Flavobacterium columnare. Syst Appl Microbiol 45:126293. doi:10.1016/j.syapm.2021.126293 35026686

[8] LaFrentz BR, Klesius PH. 2009. Development of a culture independent method to characterize the chemotactic response of Flavobacterium columnare to fish mucus. J Microbiol Methods 77:37–40. doi:10.1016/j.mimet.2008.12.011 19166883

[9] Hofer C, Lingenhag S, Serra Moncadas L, Rain-Franco A, Andrei AS. 2023. Q20+ Nanopore sequencing data recover a high-quality Asticcacaulis sp. genome. Microbiol Resour Announc 12:e0071723. doi:10.1128/MRA.00717-23 37772872 PMC10586169

[10] Kolmogorov M, Yuan J, Lin Y, Pevzner PA. 2019. Assembly of long, error-prone reads using repeat graphs. Nat Biotechnol 37:540–546. doi:10.1038/s41587-019-0072-8 30936562

[11] Wood DE, Lu J, Langmead B. 2019. Improved metagenomic analysis with Kraken 2. Genome Biol 20:257. doi:10.1186/s13059-019-1891-0 31779668 PMC6883579

[12] Samaha G, Samsing F, O’Brien M, Jaya F. 2025. ONT-bacpac-nf (Version 1.0.0) [Computer software]. 10.48546/workflowhub.workflow.1263.1.

[13] Churchman EM, Parello G, Lange MD, Farmer BD, LaFrentz BR, Beck BH, Liles MR. 2022. Draft genome sequences of Flavobacterium covae strains LSU-066-04 and LV-359-01. Microbiol Resour Announc 11:e0035222. doi:10.1128/mra.00352-22 35703564 PMC9302162

[14] Goris J, Konstantinidis KT, Klappenbach JA, Coenye T, Vandamme P, Tiedje JM. 2007. DNA-DNA hybridization values and their relationship to whole-genome sequence similarities. Int J Syst Evol Microbiol 57:81–91. doi:10.1099/ijs.0.64483-0 17220447

[15] Aziz RK, Bartels D, Best AA, DeJongh M, Disz T, Edwards RA, Formsma K, Gerdes S, Glass EM, Kubal M, et al.. 2008. The RAST Server: rapid annotations using subsystems technology. BMC Genomics 9:75. doi:10.1186/1471-2164-9-75 18261238 PMC2265698

[16] Tatusova T, DiCuccio M, Badretdin A, Chetvernin V, Nawrocki EP, Zaslavsky L, Lomsadze A, Pruitt KD, Borodovsky M, Ostell J. 2016. NCBI prokaryotic genome annotation pipeline. Nucleic Acids Res 44:6614–6624. doi:10.1093/nar/gkw569 27342282 PMC5001611

[17] Tamura K, Stecher G, Kumar S. 2021. MEGA11: molecular evolutionary genetics analysis version 11. Mol Biol Evol 38:3022–3027. doi:10.1093/molbev/msab120 33892491 PMC8233496

[18] Chokmangmeepisarn P, Thangsunan P, Kayansamruaj P, Rodkhum C. 2021. Resistome characterization of Flavobacterium columnare isolated from freshwater cultured Asian sea bass (Lates calcarifer) revealed diversity of quinolone resistance associated genes. Aquaculture 544:737149. doi:10.1016/j.aquaculture.2021.737149

[19] Nguyen D, Chokmangmeepisarn P, Khianchaikhan K, Morishita M, Uchuwittayakul A, LaFrentz B, LaFrentz B, Rodkhum C. 2025. Comparative genomic analysis of Flavobacterium species causing columnaris disease of freshwater fish in Thailand: insights into virulence and resistance mechanisms. BMC Veterinary Research. 10.1186/s12917-025-04488-3 PMC12087154 40389923

[20] Watts JEM, Schreier HJ, Lanska L, Hale MS. 2017. The rising tide of antimicrobial resistance in aquaculture: sources, sinks and solutions. Mar Drugs 15:158. doi:10.3390/md15060158 28587172 PMC5484108

